# Comparison of Classical Kinematics, Entropy, and Fractal Properties As Measures of Complexity of the Motor System in Swimming

**DOI:** 10.3389/fpsyg.2016.01566

**Published:** 2016-10-07

**Authors:** Tiago M. Barbosa, Wan X. Goh, Jorge E. Morais, Mário J. Costa, David Pendergast

**Affiliations:** ^1^Physical Education and Sport Science Academic Group, Nanyang Technological UniversitySingapore, Singapore; ^2^CIDESD - Research Centre in Sports, Health and Human DevelopmentVila Real, Portugal; ^3^Department of Sport Science, Polytechnic Institute of BragançaBragança, Portugal; ^4^Department of Sport Science, Polytechnic Institute of GuardaGuarda, Portugal; ^5^Department of Physiology and Biophysics, University at BuffaloNew York, NY, USA

**Keywords:** swimming, non-linear parameters, variability, predictability, complexity, human movement

## Abstract

The aim of this study was to compare the non-linear properties of the four competitive swim strokes. Sixty-eight swimmers performed a set of maximal 4 × 25 m using the four competitive swim strokes. The hip's speed-data as a function of time was collected with a speedo-meter. The speed fluctuation (*dv*), approximate entropy (*ApEn*) and the fractal dimension by Higuchi's method (*D*) were computed. Swimming data exhibited non-linear properties that were different among the four strokes (14.048 ≤ *dv* ≤ 39.722; 0.682 ≤ *ApEn* ≤ 1.025; 1.823 ≤ *D* ≤ 1.919). The *ApEn* showed the lowest value for front-crawl, followed by breaststroke, butterfly, and backstroke (*P* < 0.001). Fractal dimension and *dv* had the lowest values for front-crawl and backstroke, followed by butterfly and breaststroke (*P* < 0.001). It can be concluded that swimming data exhibits non-linear properties, which are different among the four competitive swimming strokes.

## Introduction

Water is a unique and challenging environment for humans who are not specially prepared to propel themselves in this environment. Competitive swimmers use one of the four swim strokes as locomotion technique. Swimming is a periodically accelerated motion (Barbosa et al., 2010):

(1) $$v=v_{0}+\Delta v(t)$$

where *v* is the subject's mean velocity, *v*_0_ is the subject's velocity at the beginning of the stroke cycle, Δ*v* is the variation of the swimming velocity over the stroke cycle, and *t* is the time. Hence, the swimmer has intra-cyclic variations of the horizontal velocity of his body, also known as “speed fluctuation” (i.e., Δ ≠ *0* m/s, otherwise it will be a uniform motion as *v*_*i*_ = *v*_0_). The mechanism underlying the accelerations and decelerations (or speed fluctuation) within each stroke is related to two external forces (propulsive force and hydrodynamic drag) acting upon the swimmer and it is an application of Newton's law of motion:

(2) $$a=\frac{F_{\mathrm{Pr}}\;+\;F_{D}}{m}$$

Where *a* is the acceleration (or speed fluctuation), *F*_*Pr*_ is the total propulsive force (in the traveling direction of displacement), *F*_*D*_ is the hydrodynamic drag force (opposite to the traveling direction), and *m* is the subject's body mass. In an acceleration-deceleration time graph, positive slopes correspond to *F*_*Pr*_ higher than *F*_*D*_, while negative slopes correspond to *F*_*Pr*_ lower than *F*_*D*_. One part of the *F*_*Pr*_ produces mechanical work to overcome *F*_*D*_ (*F*_*D*_ = *k*^.^ *v*^2^), so:

(3) $$w_{Fd}=\int_{0}^{T}k{\left[v_{0}+\Delta v(t)\right]}^{3}dt$$

Where *w*_*Fd*_ is the mechanical work, *k* is the drag factor (k = 0.5 ^.^ ρ^.^ S ^.^ *C*_*D*_; fluid density, surface area and drag coefficient, respectively), *v* is the subject's mean velocity, *v*_0_ is the subject's velocity at the beginning of the stroke cycle, Δ*v* is the variation of the swimming velocity over the stroke cycle, and *t* is the time. These changes within each stroke cycle can be tested by classical biomechanics parameters (i.e., linear parameters).

There is a solid body of knowledge describing speed fluctuation in human swimming by the coefficient of variation (*dv*) (Barbosa et al., 2005). The speed fluctuation is a parameter that quantifies the variations of the instantaneous speed around the mean speed in a full stroke cycle. The *dv* is the most cited variable and was first introduced by Barbosa et al. (2005). Others have suggested assessing the difference between maximal and minimal speed (Figueiredo et al., 2012a) or ratios between maximal and minimal speed with average speed (Psycharakis and Sanders, 2009) to assess the speed fluctuation. The *dv* is highest in the Breaststroke, followed by the Butterfly, Backstroke and Front-crawl, respectively (Barbosa et al., 2010). The *dv* is also related to the energy cost of locomotion. It is often used as an estimation of the energy cost of swimming and/or swimming efficiency (Barbosa et al., 2005). Higher the *dv*, higher the energy cost and hence, poorer the swimming efficiency.

In a linear system, a small change in one input has a proportional and quantifiable change in the output. As far as human movement concerns (and notably in elite sports), this may not always be the case. Sometimes small changes in the input are not reflected in the variables selected to monitor one's motor behavior. In such event, non-linear parameters are quite useful because they exhibit a very sensitive dependence on the inputs. Non-linear complex dynamical systems are characterized by interaction-dominant dynamics, which is at odds with component dominance and with additive effects. In elite sports, practitioners bridge these concepts to the marginal gains “theory.” The latter encompasses the rationale that it is the sum of very small changes (each one of them might be non-significant) that helps the elite athlete to excel. It is hypothesized that such small changes can be monitored by non-linear parameters. In the motor control of a biological system, the variables playing a role on a main outcome are not independent. There is an interplay among several variables that ultimately will affect the main outcome. Therefore, one may reason that each marginal gain will trigger a change in the interplay among the components of the system affecting ultimately the main outcome. Under complex science it is more accurate to note that rather than the sum of trivial changes, it is the dynamic interaction in play that may help to excel. Each trivial change that a practitioner may point out might indeed be a change in the dynamic interaction of the systems' components though.

Academics with research interest on these topics, note that the constraints-led approach is an interesting framework to be considered (Davids et al., 2008). It is assumed that the variability and complexity of the motor behavior depend on the role played by environmental, task, and organismic constraints. The level of complexity can be due to different constraints acting upon the performer. On top of that, an advantage of this approach is that it encompasses main features of non-linear complex dynamical systems. It is not only the constraints acting on the system but on top of that, how such constraints interact and interplay to deliver a given outcome. Swimming patterns (as the case of the speed-time series in swimming reported early on) may be complex and sometimes linear parameters do not provide insightful details. Hence, the question to be raised is if non-linear parameters can enlighten us. Thus, far, only a few articles can be found in the literature that assessed the complexity of motor behavior in competitive sports by entropy and fractal properties.

The entropy is an informational non-linear parameter that describes the degree of irregularity/complexity inherent to the order of the elements in a time-series (Bravi et al., 2011). Approximate entropy (*ApEn*) is a dimensionless measure that ranges between 0 (signifying repeatability) and 2 (signifying randomness) (Pincus, 1991). *ApEn* has been selected for the analysis of time-series data in postural balance (Kee et al., 2012) and gait analysis (Arif et al., 2004). The *ApEn* is higher for elderly than young counterparts at selected gait paces (Arif et al., 2004). As far as competitive sport is concerned, the complexity of the motor behavior has been reported in climbing (Cordier et al., 1994) and football (Duarte et al., 2013). At least one research assessed the entropy of the heart rate response in swimming (Merati et al., 2015). Entropy was also reported for speed-time series of young swimmers over a full season (Barbosa et al., 2015). *ApEn* decreased as a function of time, with greater decreases at the end of the season (Barbosa et al., 2015). Hence, entropy seems capable of discriminating swimming performance and enlightening on one's technique. Entropy can provide insight on the inter-cyclic variability of the swim strokes; i.e., the degree of repeatability/randomness of the stroke cycles over a lap or an event. If so, one may wonder if there are differences when the four competitive swim strokes are compared to each other. This can provide insight on the factors (i.e., system components) interplaying in each swim stroke and ultimately help high-level swimmers to excel in a sport where performances are differentiated to 0.01 s.

Fractal dimension is categorized as an invariant non-linear parameter describing the properties of a system that demonstrates fractality or other properties that do not change over time and/or space (Bravi et al., 2011). Fractal analysis has recently been applied to study a wide range of objects and systems in Biology and Medicine (Havlin et al., 1995; Liu et al., 2003; Tan et al., 2009; West, 2013). In human gait the subject will either “accelerate” or “decelerate” his speed for a given unit of measure in each point of time (Havlin et al., 1995). A fractal landscape can be noted for the speed-time series (Peng et al., 1992). Fractal quantification is based on unconventional views of scaling and dimension. For both self-similar and self-affine series, such as time series, it can be generalized as (De Santis, 1997):

(4) $$M(\delta )\propto \delta^{d-D}$$

Where *M* is the typical measure of the analyzed *d*-dimensional data, δ is the linear ruler and *D* is the fractal dimension. A profile is said to be self-similar when it scales in the same way in both x and y coordinates of the plane and *D* is a self-similar dimension; whereas if one measure of the vertical range spanned over the horizontal range, the profile is self-affine (De Santis, 1997). The variable that is selected to assess the fractal properties of biological phenomena is the “fractal dimension” (*D*). As a rule of thumb if: (i) *D* = 0, there are 0-dimensional sets; (ii) *D* = 1, there are 1-dimensional sets (i.e., length only, straight line); (iii) *D* = 2, there are 2-dimensional sets (i.e., length x width, surface) and; (iv) *D* = 3, there are 3-dimensional sets (i.e., length x width x height, volume). All in all, as the *D* value increases, likewise for the complexity of the time-series. The fractal proprieties of human gait on land have been reported a few times in the literature (Sekine et al., 2002; Schiffman et al., 2009). For instance, fractal dimension decreases over time when walking for 2 h carrying a backload (Schiffman et al., 2009). Fractal dynamics in competitive sports has been reported for running (Hoos et al., 2014), rowing (Den Hartigh et al., 2015), and cycling (Tucker et al., 2006). Neumeister et al. (2004) reported that the *D* for fishes was 1.62. Considering what was reported in the literature for humans on land and fish in water, fractal dimension is expected to range from 1.0 to 2.0 in competitive swimming. There is only one paper reporting the case study for one single breaststroker (Witte and Blaser, 2001). The authors noted a *D* between 1.45 and 1.75 for this swimmer. To the best of our knowledge, the fractal dimension of the four swim strokes has never been compared before. In fact, past research have largely focused on quantitative (i.e., classical kinematics) and qualitative data (i.e., graphs depicting the typical curves) for speed-time series in swimming (Craig et al., 2006; Figueiredo et al., 2009; Psycharakis and Sanders, 2009). Since the interaction and complexity of the system components happen at different levels and time scales, there are *D* variations for different locomotion and sport techniques. As a result, this same rationale may underpin the hypothesis that the fractal properties of the four swim strokes should be different as well.

The aim of this research was to compare the non-linear properties of the four swim strokes. It was hypothesized that like other locomotion techniques, swimming will exhibit non-linear proprieties, including the entropy and fractal dynamics. However, because of the different configurations of constraints acting on the swim strokes, there will be differences among the four.

## Material and methods

### Subjects

Sixty-eight high-level swimmers were assessed (34 males: 17.06 ± 4.11 years old; 34 females: 14.97 ± 2.96 years old). The sample included age-group national record holders, age-group national champions, and other swimmers that compete on regular basis at national or international competitions.

Coaches, parents or guardians, and the swimmers gave informed written consent/assent for participation in this study. All procedures were in accordance with the Helsinki Declaration regarding human research. The University IRB also approved the research design.

### Procedures

#### Protocol

The swimmers did a standard warm-up of 1500 m including continuous swimming at low-moderate intensity, with specific drills and sprints at the end. Each swimmer undertook a set of all-out (i.e., maximal bouts) 4 × 25 m swims using randomly assigned Front-crawl or Backstroke or Breaststroke or Butterfly strokes. Swims started with a push-off and there was a 30 min rest between trials. Participants performed each trial alone with no other swimmer in the lane or nearby lanes to reduce drafting and pacing effects, and extra drag force due to exogenous factors. The swimmers were advised to start swimming after the push-off, minimize the gliding, and dolphin kicking.

#### Data collection

A speedo-meter cord (Swim speedo-meter, Swimsportec, Hildesheim, Germany) was attached to the swimmer's hip (Barbosa et al., 2013). The speedo-meter was placed on the forehead-wall of the swimming pool. A software interface in LabVIEW® (v. 2009) was used to acquire (*f* = 50 Hz), display, and process speed-time data for each trial. Data were transferred from the speedo-meter to the software by a 12-bit acquisition card (USB-6008, National Instruments, Austin, Texas, USA). Thereafter, data were exported to signal processing software (AcqKnowledge v. 3.9.0, Biopac Systems, Santa Barbara, USA) and filtered with a 5 Hz cut-off low-pass 4th order Butterworth filter, according to the analysis of the residual error vs. cut-off frequency output. Push-off start, dolphin kicks and the finish were discarded in the follow-up analysis. To run some of the non-linear parameters, at least 500 speed-time pairs are recommended to be collected (Yentes et al., 2013). As far as we understand, the speedo-meter is the most convenient device to do so, when benchmarked with other equipment available (e.g., motion-capture systems or inertia measurement units).

##### Speed fluctuation

The intra-cyclic variation of the horizontal velocity of the hip (i.e., *dv*) was analyzed as previously reported (Barbosa et al., 2005, 2010):

(5) $$dv\;=\;\frac{\sqrt{\frac{\sum_{i}(v_{i}-{\bar{v)}}^{2}.\;F_{i}}{n}}}{\frac{\sum_{i}v_{i}.\;F_{i}}{n}}\cdot 100$$

Where *dv* is the intra-cyclic variation of the horizontal velocity of the hip, $\overset{\_}{v}$ is the mean swimming velocity, *v*_*i*_ is the instant swimming velocity, *F*_*i*_ is the acquisition frequency, and *n* is the number speed-time pairs. The *dv* mean values of three consecutive stroke cycles between the 11th m (i.e., removing the effect of the start) and 24th m (removing the effect of the finish) from the starting wall were considered for further analysis.

##### Approximate entropy

The *ApEn* was computed based on the Pincus' algorithm (Pincus, 1991):

(6) $$ApEn(N,m,r)\;=\;\ln\left[\frac{C_{m}(r)}{C_{m+1}(r)}\right]$$

Where *ApEn* is the approximate entropy, *N* is the data length [*N* = 700 speed-time pairs, as suggested by Yentes et al. (2013)], *m* is the embedding dimension (*m* = 2, because two consecutive cycles contributing to two data points were considered for each mobile window), *r* is the tolerance value or similarity criterion [*r* = 0.1, determined beforehand as the maximum *ApEn* for a wide range of *r* values between 0.01 and 0.3 as suggested by others (Chon et al., 2009; Yentes et al., 2013)], and:

(7) $$C_{im}(r)\;=\;\frac{n_{im}}{N-m+1}$$

Where *C*_*im*_ is the fraction of patterns of length, *n*_*im*_ is the number of patterns that are similar between two sets (given the similarity criterion, *r*), *N* is the data length, and *m* is the embedding dimension. *ApEn* is reported quite often in the literature because it requires a low computational demand and is less affected by noise. However, *ApEn* is heavily dependent on the record length and lacks relative consistency. If *ApEn* of one dataset is higher than another, it might not remain higher for all conditions tested (Richman and Moorman, 2000). To mitigate this concern, the data length was kept constant. Richman and Moorman (2000) compared *ApEn* with Sample Entropy. The latter was proposed to overcome the limitations by the former parameter. The authors assessed *ApEn* and sample entropy for a wide range of *N* (0 ≤ *N* ≤ 1000) and *r* (0.01 ≤ *r* ≤ 1). When *N* = 700, *m* = 2, and *r* = 0.1, as set for this research, the bias between *ApEn* and sample entropy was minimal.

##### Fractal dimension

There are two main algorithms reported in the literature to compute the *D*, the box-counting (Block et al., 1990) and the Higuchi's methods (Higuchi, 1988). In other scientific fields, such as Medicine (e.g., medical imaging or neurophysiology), Biology or Tomography, the box-counting method seems to be reported on a regular basis assessing the bodies' geometries (Liu et al., 2003); while the Higuchi's method is mostly used for time-series analysis (Sourina et al., 2010; Wang et al., 2011). The latter method is the most suitable for this research involving time-series (Castiglioni et al., 2011). Hence, the *D* was calculated by the Higuchi's methods (Higuchi, 1988):

(8) $$D\;=\;\frac{d\log N\left(L\left(k\right)\right)}{d\log\left(k\right)}$$

Where *D* is the fractal dimension, *N* is the number of new points from the speed-time series and *k* is the scaling factor. In other scientific fields, researchers have been assessing the fractal properties with different algorithms.

### Statistical analyses

Data normality was tested by the Shapiro–Wilk test. Data is described as mean ± 1 *SD* (i.e., 68.27% confidence interval) and 95% of confidence interval (95 CI).

Repeated measures (within-subjects' ANOVA) analysis was performed to compare the four swimming strokes (*P* ≤ 0.05). This analysis was followed-up by multivariate tests (within-subjects' ANCOVA) to examine the role of potential confounding factors such as sex and swim speed (Δ*v*) (*P* ≤ 0.05). The Δ*v* was set as the speed amplitude (i.e., maximum minus minimum speeds in the four trials). Whenever needed, analyses of the variations were followed up by Bonferroni tests (*P* ≤ 0.05). Effect size was computed based on eta-squared (η^2^) and interpreted as: Without effect if 0 < η^2^ ≤ 0.04; minimum if 0.04 < η^2^ ≤ 0.25; moderate if 0.25 < η^2^ ≤ 0.64 and; strong if η^2^> 0.64. Cohen's *d* was also computed for the *post-hoc* testing of pairwise swim strokes for significant ANOVAs. Effect sizes were considered as small (*d* = 0.2), moderate (*d* = 0.5), and large (*d* = 0.8). In sport science research that assesses elite athletes, the effect sizes are interpreted differently, whereby an effect size above 0.2 is already considered as having a true impact on the performance while above 0.5 reflects a very meaningful impact (Buchheit, 2016).

## Results

Analysis across the four strokes returned significant variations with moderate-strong effects in all variables [*dv*: *F*_(3, 201)_ = 596.498, *P* < 0.001; η^2^ = 0.89; *ApEn*: *F*_(3, 201)_ = 89.074, *P* < 0.001; η^2^ = 0.57; *D*: *F*_(3, 201)_ = 61.112, *P* < 0.001; η^2^ = 0.47] (Table 1). *Post-hoc* analysis showed that *dv* is significantly different for all pairs (*P* < 0.001) except between front-crawl and backstroke. The *ApEn* was also significantly different among all swim strokes (*P* < 0.001) except the front-crawl vs. breaststroke, which had a small effect size (*P* = 0.21; *d* = 0.40). The *D* was significantly different among all swim strokes (*P* < 0.001) except the front-crawl vs. backstroke pair (*P* = 0.98; *d* = 0.16).

**Table 1:** **Analysis of the variations across the four swimming strokes**.

	**Front-crawl Mean ± 1 *SD*(95 CI)**	**Backstroke Mean ± 1 *SD*(95 CI)**	**Breaststroke Mean ± 1 *SD*(95 CI)**	**Butterfly Mean ± 1 *SD*(95 CI)**	***F***	***P***	**η^2^**
*dv* [%]	14.04 ± 4.65 (12.90;15.12)	13.44 ± 3.42 (12.60;14.26)	39.72 ± 4.81 (38.55;40.88)	25.62 ± 4.16 (24.61;26.62)	596.498	<0.001	0.89
*ApEn* [dimensionless]	0.68 ± 0.15 (0.64;0.71)	1.03 ± 0.19 (0.97;1.07)	0.73 ± 0.10 (0.70;0.75)	0.85 ± 0.17 (0.81;0.89)	89.074	<0.001	0.57
*D* [dimensionless]	1.84 ± 0.08 (1.23;1.26)	1.82 ± 0.07 (1.24;1.26)	1.92 ± 0.03 (1.31;1.36)	1.88 ± 0.07 (1.30;1.34)	61.112	<0.001	0.47

To examine the effects of sex and speed as potential confounding and interacting factors, multivariate analysis was computed (Table 2). For all variables studied, the effects and interactions were non-significant and without or minimum effect sizes (0.05 ≤ η^2^ ≤ 0.34). Hence, the sex and the range of speeds swam did not have an effect on the data.

**Table 2:** **Multivariate test, controlling the effect of sex, and speed on the selected variables**.

	**Speed effect**					**Sex effect**					**Speed** × **Sex interaction**				
	**Λ_Pillai's_**	**Λ_Wilk's_**	***F***	***P***	**η^2^**	**Λ_Pillai's_**	**Λ_Wilk's_**	***F***	***P***	**η^2^**	**Λ_Pillai's_**	**Λ_Wilk's_**	***F***	***P***	**η^2^**
*dv*	0.069	0.931	1.553	0.21	0.010	0.040	0.960	0.884	0.45	0.005	0.109	0.893	1.233	0.30	0.016
*ApEn*	0.052	0.948	1.151	0.34	0.007	0.061	0.939	1.370	0.26	0.010	0.096	0.906	1.07	0.38	0.015
*D*	0.023	0.977	0.484	0.69	0.008	0.053	0.947	1.164	0.33	0.010	0.096	0.906	1.07	0.38	0.034

## Discussion

The aim of this study was to compare the non-linear properties of the four competitive swim strokes. As hypothesized, swimming does exhibit non-linear properties that are different among the four swimming strokes (0.68 ≤ *ApEn* ≤ 1.03; 1.82 ≤ *D* ≤ 1.92). The *ApEn* showed the lowest value in front-crawl, followed by the breaststroke, butterfly, and backstroke. Fractal dimension had the lowest values in front-crawl and backstroke, followed by butterfly and breaststroke.

The *dv* was selected as a classical kinematics variable for this study (i.e., linear parameter). The *dv* is considered a well-rounded parameter to bring insight on the efficiency and energy cost of swimming (Barbosa et al., 2005, 2010; Figueiredo et al., 2012b), as well as, inter-limb coordination (Leblanc et al., 2007; Schnitzler et al., 2008). In other words, the *dv* represents a balance between propulsive (thrust) and resistive forces (Equation 2). It is used as an efficiency estimator because the energy expenditure will be related to the mechanical work done by the swimmer (Equation 3). The *dv* was significantly different for all stroke pairs tested except between front-crawl and backstroke. Hence, the lowest *dv* was found for the front-crawl and backstroke, followed by butterfly and finally breaststroke. The *dv* observed for the four swim strokes is related to several factors. Papers comparing the energetics, kinematics or kinetics of the four swim strokes can be found and all report the same trend for the various strokes. The energy expenditure (Barbosa et al., 2006), tethered swimming force (Morouço et al., 2011), drag force (Kolmogorov et al., 1997), and intra-cyclic mechanical impulse (Barbosa et al., 2010) show the highest values in breaststroke, followed then by butterfly, backstroke and front-crawl. Data suggests that swim strokes producing higher tethered forces and drag forces will impose higher intra-cyclic mechanical impulse, hence larger *dv* and ultimately more energy expenditure. Overall, the data collected in this study is in tandem with previous reports. This agreement between the present and previous studies give confidence that the findings for remaining variables determined in the present study are not affected by the participants recruited, data collection procedure, raw data obtained, or data analysis.

The selection of multiple non-linear measures (for this research the *ApEn* and *D*), as well as the inclusion of a linear measure (i.e., classical kinematics, the *dv*), can enhance the behavioral discriminations (Neumeister et al., 2004). The *ApEn* falls under the category of informational parameters, *D* in the invariant category and *dv* in the statistical domain (Bravi et al., 2011). The *ApEn* enables one to learn how deterministic the motor behavior is. In periodic motions, like swimming, it can provide insight on the inter-cyclic variations as a function of swim time. A low *ApEn* suggests that the time-series is deterministic and a high value indicates randomness. The *ApEn* showed the lowest value (i.e., very determinist; *ApEn* = 0.68 ± 0.15) for front-crawl followed by the breaststroke, butterfly, and backstroke (i.e., fairly deterministic; *ApEn* = 1.03 ± 0.19). To our knowledge, only one paper reported *ApEn* in human swimming. They reported a decrease from 0.610 (95 CI: 0.544–0.676) at beginning to 0.580 (95 CI: 0.530–0.631) at the end of the season in young swimmers (Barbosa et al., 2015). Based on these data, it seems that *ApEn* might be different according to expertise levels and/or age of the participants. Support for this comes from other scientific fields, where entropy was related to sensory inputs, motor control mechanisms, and biomechanical behavior (Arif et al., 2004; Kee et al., 2012; Menayo et al., 2014). These authors noted that the entropy is different depending on the subject's level of expertise. One can suggest that the same factors could affect human swimming. The coordination between system components occurs at several levels and time scales leading to a high complexity. This can help to explain why backstroke showed the highest *ApEn* and front-crawl the lowest. The supine position (changing the information obtained by several sensory inputs such as vision and vestibular systems) concurrent with the challenge of synchronizing four alternated limbs (two upper-extremities that must perform each one three to four propulsive phases underwater plus two lower-limbs that must do within the stroke cycle six kicks) and the full body rotation (to each side of the body in the longitudinal axis) are reasons enough to propose that these backstroke features lead to a higher *ApEn*. However, follow-up studies are needed for a deeper insight on the mechanisms explaining the *ApEn* in swimming. All in all, as reported in the literature, more difficult or less mastered tasks reveal more random patterns of variation (Wijnants et al., 2009).

The value of *D* can provide information on the complexity and irregularity of the time-series data. The *ApEn* analysis is often complemented with the analysis of *D* (Tan et al., 2009). The higher the *D*, the more complex the time series is. Human swimming showed fractal properties (1 ≤ *D* ≤ 2). Gold fish was reported to have a *D* ~1.62 (1.29 ≤ *D* ≤ 1.74) (Neumeister et al., 2004) and human gait on land 1.12 ≤ *D* ≤ 1.43 (Schiffman et al., 2009). Running at half-marathon pace, the *D* was 1.70 ± 0.10 (Hoos et al., 2014). In rowing, the *D* was 1.22 ± 0.03 for highly-skilled participants, whereas it was 1.30 ± 0.03 for lower-skilled counterparts. This corresponds to a value of 1.2 for optimal complexity (1.1 = regular Brownian noise; 1.5 = random white noise) (Den Hartigh et al., 2015). We found values ranging between 1.82 ≤ *D* ≤ 1.92. Therefore, human swimming is a locomotion technique that exhibits fractal proprieties. Compared to other forms of locomotion; fish in water and human on land; human swimming shows a higher complexity. While fishes are fully adapted to aquatic environment, humans are adapted to land. We are unaware of research comparing the four swim strokes or assessing one single swim stroke in a sample of swimmers. The case study of a breaststroker can be found in the literature and the authors noted a *D* ranging between 1.75 and 1.45 with increasing swim paces (Witte and Blaser, 2001). However, no further details are shared on the algorithm selected to compute the parameter. Comparing the four swim strokes, front-crawl, and backstroke had the lowest *D*, followed by butterfly and breaststroke.

The degree of complexity exhibited by each swim stroke can depend on several constraints experienced by the subjects. The constraint-led approach by Davids et al. (2008) encompasses features of the “non-linear dynamical systems” as shared early on. This approach provides a framework, combining a balanced interaction between organismic, environmental and task constraints. Environmental constraints might include the drag force acting upon the subject in different strokes. We do know that the amount of resistance is higher at breaststroke, followed by butterfly, backstroke, and front-crawl (Kolmogorov et al., 1997). The task constraints can be related to the selected combination of stroke rate-stroke length for each stroke. Several papers can be found in the literature reporting such relationships (e.g., Barbosa et al., 2010). The organismic constraints might be due to specific anthropometric features that are more suitable for a given swim stroke than another, or the performance level. The interaction of these three constraints can explain the differences in complexity observed among the four swim strokes.

This study provides new insights into swim analysis, whereby non-linear properties differentiate the four swim-strokes. These results encourage the use of non-linear properties to analyze swimming beyond the traditional methods. Future research on this topic should focus on examining: (i) if these non-linear properties change according to the expertise level of the subjects recruited; (ii) if human swimming does exhibit non-linear properties, future research should focus on the understanding of the mechanisms underpinning such phenomenon (e.g., how the constraints-led perspective or a model of self-organized criticality, interaction dominant dynamics, or degeneracy can explain the complexity of the motor behavior).

It can be concluded that swimming data exhibits non-linear properties, which are different among the four competitive swimming strokes. The *ApEn* and *D* can provide insight on the inter- and intra-cyclic changes of the stroke cycles in swimming. The *ApEn* showed the lowest value at front-crawl, followed by the breaststroke, butterfly, and backstroke. The *D* shows the lowest values at front-crawl and backstroke, followed by butterfly and then breaststroke.

## Author contributions

Conceived and designed the experiments: TB, MC, DP. Performed the experiments: WG. Analyzed the data: TB, WG, JM, Draft the manuscript: TB, WG, JM, MC, DP.

### Conflict of interest statement

The authors declare that the research was conducted in the absence of any commercial or financial relationships that could be construed as a potential conflict of interest.
